# Y6 and its derivatives: molecular design and physical mechanism

**DOI:** 10.1093/nsr/nwab121

**Published:** 2021-07-14

**Authors:** Qingya Wei, Jun Yuan, Yuanping Yi, Chunfeng Zhang, Yingping Zou

**Affiliations:** College of Chemistry and Chemical Engineering, Central South University, China; College of Chemistry and Chemical Engineering, Central South University, China; Beijing National Laboratory for Molecular Sciences, CAS Key Laboratory of Organic Solids, Institute of Chemistry, Chinese Academy of Sciences, China; National Laboratory of Solid State Microstructures, School of Physics, and Collaborative Innovation Center for Advanced Microstructures, Nanjing University, China; College of Chemistry and Chemical Engineering, Central South University, China

## Abstract

Y6 and its derivatives have advanced the efficiency of organic solar cells to 15%-18%. This perspective reveals the device and photo physics features of Y-series based devices and proposed some guidelines for future molecular design.

Non-fullerene-acceptor (NFA) materials in the bulk heterojunction (BHJ) play an important role in high-performance organic solar cells (OSCs). Recently, Y-series acceptors with an A-DA′D-A structure have been a research hotspot and advanced device efficiency to 15%–18%. This perspective will focus on the discovery of Y6 and its derivatives as well as the intrinsic physical mechanism of Y-series-based devices, which provides a guideline for future molecular design.

As the original molecule of the Y-series acceptors, BZIC (Y0) consists of a benzotriazole central core and a pentacyclic fused backbone with a simple synthesis process of Stille coupling, double intramolecular Cadogan reductive cyclization, N-alkylation, nucleophilic reaction and Knoevenagel condensation [1]. After expanding the conjugated framework, attaching the terminal alkyl chains, replacing the electron-deficient A′ core and fluorinating the end groups, a well-known Y6 was synthesized with an outstanding photovoltaic performance exceeding 15% [2]. In terms of molecular structure, the reason why Y6 performs well can be summarized as the following. Differently from the electron-donating fused backbone of ITIC, Y6 adopts a fused donor-acceptor-donor (DA′D) framework, fluorinated end groups and a curved geometry, which enables Y6 to have more π–π stacking possibilities and stronger near-infrared absorption, therefore, the intermolecular stacking, optical absorption and charge transfer properties are enhanced. Meanwhile, the non-radiative recombination of Y-series acceptors is suppressed due to the strengthened photoluminescence quantum yield, which could result from the electron-deficient A′ and the molecular geometry.

Subsequently, substantial efforts have been paid to optimization of Y6 in the side chains, backbone and end groups (Fig. 1a). Zou and Yan *et al.* regulated the branched position of 2-ethylhexyl in the pyrrole of Y6 away from the nitrogen atoms to obtain N3 and N4 with increasing solubility. Using PM6 as the donor, the PM6 : N3 blend showed the best molecular orientation and domain size, resulting in the highest device efficiency [3]. Meanwhile, Sun *et al.* introduced branched alkyl chains with different lengths to the thiophene β-position of Y6. As a result, the molecular stacking behavior was improved and an excellent efficiency of 18.32% with a high fill factor (FF) of 81.5% was achieved [4]. These works revealed the importance of branching positions and the length of bulky side chains in NFAs. As for the backbone reformation, the asymmetric strategy and introduction of heteroatoms are the most popular methods. Selenium atoms were used to replace the sulfur atoms of the benzothiadiazole (BT) or thieno[3,2-b]thiophene (TT) units in Y6 to synthesize Y6Se and CH1007. These two selenium heterocyclic electron acceptors possessed redshifted absorption of ∼950 nm and achieved a high *J*_SC_ near 28 mA cm^–2^ and power conversion efficiency (PCE) over 17% [5,6]. Various terminal groups were also employed to modulate the optical and electrochemical properties of the Y-series acceptors. Halogenation was a simple but efficient method. For instance, Yan *et al.* altered the chlorine and bromine positions on the terminal group to synthesize the BTP-ClBr. Enhanced device performance, especially the *V*_OC_ and FF, were achieved due to the shallower LUMO energy level and the improved blend morphology [7]. In 2017, Li *et al.* proposed a smart strategy of polymerized small molecular acceptors (PSMAs), which was very important for the development of all-polymer OSCs [8]. Using Y-series acceptors as key building blocks combined with Li's strategy, the PSMAs have boosted the efficiency of all-polymer OSCs by over 17% [9].

**Figure 1.**
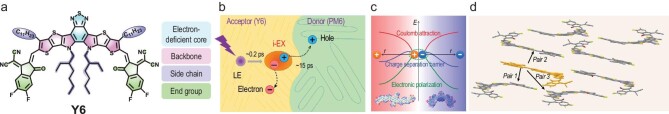
(a) The molecular structure of Y6 and molecular modification for its analogs. (b) Schematic diagram of the i-EX state formed in the neat Y6 domain as an intermediate state to separate local excitation (LE) states into free polarons. Adapted with permission from ref. [10]. Copyright 2020 American Chemical Society. (c) Schematic description of polarization-assisted charge separation. Adapted with permission from ref. [12]. Copyright 2020 American Chemical Society. (d) Molecular pairs in the Y6 single crystal.

In addition to the above representative examples in the material design, the intrinsic mechanism of Y-series acceptors was also studied, revealing the reason for the outstanding device performance. Zhang *et al.* investigated the charge separation mechanism in PM6 : Y6 blend using a method of broadband transient absorption (TA) spectroscopy. They demonstrated an intra-moiety excited (i-EX) state that formed in the neat Y6 domain instead of the charge-transfer (CT) state at the PM6/Y6 interfaces, consequently enabling dissociation of excitons into free holes and electrons. Hence, manipulating the interplay between intra-moiety and interfacial excited species in polymer/NFA blends could provide a promising strategy for future improvement of the charge separation (Fig. 1b) [10]. Furthermore, Zhu *et al.* calculated the exciton binding energy (*E*_b_) in neat Y6 to be an extremely small −0.11–0.15 eV, and further proved that the energy barrier for exciton dissociation into free charge carriers was lower than the thermal energy at room temperature, therefore, direct charge generation could be enabled without the help of D/A interfaces upon illumination [11]. Yi *et al.* also demonstrated a barrierless charge separation in Y6-based OSCs that was due to the increase in polarization energies of hole and electron during their separation that could overcome the Coulomb attraction of the interfacial CT state. The large polarization energies could be attributed to the fluorination of end groups and the incorporation of an electron-deficient A′ core in Y6 (Fig. 1c) [12]. Neher *et al.* demonstrated an efficient photocurrent generation in PM6 : Y6 under a small driving energy for dissociation of CT states. This was because an electrostatic bias potential was provided by the large quadrupolar moment of Y6 and its dimerization in a unit cell to make up for the Coulomb binding of the CT states [13]. Meanwhile, Huang *et al.* demonstrated the generation of triplet excitons in Y6 and its analogs, and the contribution of triplet states to the device performance was demonstrated by magneto-photocurrent and transient spectroscopy [14].

More importantly, the single-crystal structure of the Y6 molecule was characterized to further understand the molecular structure–properties relationship. Liu *et al.* identified that the alkyl side chains of Y6 onto the N-atoms could greatly affect the stacking of adjacent molecules, which is totally different from that of typical ITIC analogs; the molecule presented a specific ‘banana’ shape and could form a twisted transport channel and slipped packing motif, therefore broadening the conjugated backbone and facilitating carrier transfer [15]. Furthermore, Yip *et al.* demonstrated a unique π–π stacking between the DA′D framework (*Pair 3*) in the Y6 single crystal as well as a common π–π stacking between end groups (*Pair 1* and *Pair 2*) resulting in an effective 3D ambipolar transport network (Fig. 1d). The existence of the π–π stacking of Y6 was observed not only in the single crystal but also in the PM6 : Y6 blend film through grazing incidence wide-angle X-ray scattering (GIWAXS) and molecular dynamics simulations. This was the reason why the PM6 : Y6 blend simultaneously showed a low voltage loss and high charge generation efficiency [16].

As the most popular and successful small molecular acceptors, Y-series NFAs have been investigated systematically in recent years. However, there are still some challenges in achieving commercialization of OSCs. First of all, since the optimal *J*_SC_ and FF have reached ∼29 mA cm^–2^ [17] and 81.5% [4], respectively, it is essential to further enhance the photoluminescence quantum yield and reduce the non-radiative recombination loss by introducing the groups with a photoluminescence property, controlling the molecular aggregation behavior for an improved *V*_OC_ and getting a balance of these three parameters for a higher PCE. Meanwhile, the intrinsic mechanism of Y-series acceptors should be further studied to predictably develop new acceptors for special applications, such as indoor photovoltaics. More urgently, preparation cost and long-term stability of OSCs should be dealt with. From the perspective of electron-accepting materials, non-fused acceptors with strong intramolecular non-covalent interactions will be one of the answers to reducing the synthesis cost while maintaining a high efficiency. Regulating the molecular aggregation, avoiding light-sensitive groups (e.g. exocyclic double bond) and enhancing the heat transfer property could improve the morphology, light and thermal stability of acceptors, and thus the OSCs.
